# Ultraviolet-visible study on acid-base equilibria of aporphine alkaloids with antiplasmodial and antioxidant activities from *Alseodaphne corneri* and *Dehaasia longipedicellata*

**DOI:** 10.1038/srep21517

**Published:** 2016-02-22

**Authors:** Azeana Zahari, Abdulwali Ablat, Noridayu Omer, Mohd Azlan Nafiah, Yasodha Sivasothy, Jamaludin Mohamad, Mohammad Niyaz Khan, Khalijah Awang

**Affiliations:** 1 Department of Chemistry, Faculty of Science, University of Malaya, 50603 Kuala Lumpur, Malaysia; 2 Institute of Biological Sciences, Faculty of Science, University of Malaya, 50603 Kuala Lumpur, Malaysia; 3 Chemistry Department, Faculty of Science and Mathematics, University of Pendidikan Sultan Idris, 35900 Tanjung Malim, Perak, Malaysia

## Abstract

The UV-vis spectra of isocorydine **1**, norisocorydine **2** and boldine **3** were studied in 2% v/v acetonitrile, at constant ionic strength (0.1 M NaCl, 35 degree Celsius). The p*K*_*a*_ values of isocorydine **1** and norisocorydine **2** were 11.75 and 12.07, respectively. Boldine **3** gave a p*K*_*a*_ value of 9.16 and 10.44. All of the alkaloids **1**–**3** were stable at physiological pH; thereby all of them will not ionize, thus permitting the basic nitrogen to be protonated and accumulated within the acidic food vacuole of *Plasmodium* via pH trapping. Subsequently, acidic food vacuoles that have been neutralized by alkaloids would result in enhancement of the antiplasmodial activity. The alkaloids showed antiplasmodial activity against *Plasmodium falciparum* and antioxidant activities; DPPH radical scavenging, metal chelating and ferric reducing power. The antioxidant properties of the alkaloids under investigation revealed that in addition to the antiplasmodial activity, the alkaloids can also prevent oxidative damage. It can be prevented by binding free heme and neutralizing the electrons produced during the *Plasmodium falciparum* mediated haemoglobin destruction in the host. Slightly basic properties of the aforementioned alkaloids, along with their antioxidant activities, are advantageous in improving the suppression of malaria infection that cause less damage to the host.

The tropical rain forest of Malaysia is one of the 12-mega biodiversity countries in the world that has not less than 15,000 species of higher plants, thus, they provide a promising source of chemical entities for biological activities^1^. Analysis of data in between 1950–1980 on prescription of drugs dispensed in the United States indicated about 25% contained plant extracts or active principle derived from higher plants^2^. Lauraceae is one of the largest and important families of higher plants throughout tropical and subtropical forests. It consists of 2,500–3,000 species in 67 genera all over the world while in Malaysia it comprises about 287 species in 16 genera^3^,^4^,^5^. Among that, genus *Alseodaphne* and *Dehaasia* have been studied for Malaysian plant in this communication. Both genera are known to be rich in isoquinoline alkaloids, particularly aporphine alkaloids that contain various interesting biological activities such as smooth muscle relaxant^6^, antibacterial, antifungal and cytotoxic activities^7^.

In 2013, World Health Organization (WHO) estimated 198 million cases reported with 584,000 deaths globally and is endemic throughout tropical and subtropical^8^. Among that, 3,850 cases reported with 14 deaths in Malaysia for malaria infection transmitted by *Anopheles leucosphyrus* mosquitoes^9^,^10^. Present drugs, chloroquine and artemisinine, have become ineffective because of the occurrence detected in 4 countries; Cambodia, Myanmar, Thailand and Vietnam that resistant to *Plasmodium falciparum*. Therefore, it is important to replace current drugs. Historically, the majority of the antimalarial drugs were derived from plants; which have provided a good source of lead for drug discovery^11^.

It is critical to understand the pharmacodynamics, pharmacokinetic and temperature dependent properties of drug substances because it will provide useful information on the transport or the binding mechanism from its point of administration to its point of action^12^. In order to obtain the full potential of an antiplasmodial drug, the determinations of its antioxidant and acid-base properties are essential. The importance of the antioxidant activity is to reverse or minimize the oxidative damage to the hosts caused by *Plasmodium* parasites from malaria infection. On the other hand, acidity constants (*K*_*a*_ values) of a reactant in a medium are useful physicochemical measurement to describe the extent of dissociation of functional groups with respect to pH. These parameters are also important in selecting appropriate acidic or basic reagents in drug delivery studies.

Parasites such as *Plasmodium* possesses acidic food vacuole, which is an important organelle involved in the digestion of the host haemoglobin. This digestion occurs at the acidic food vacuole which contains hemazoin. Basic natural products such as alkaloids are helpful in inhibiting the formation of hemazoin by reducing its acidity. In order to determine the acid dissociation constant of these alkaloids, the UV-vis spectroscopic method is used due to its high sensitivity (detection limits can be reached with concentrations of substances as low as 10^−6 ^M). Moreover, isoquinoline alkaloids fulfilled the important requirements of this method, which requires it to have a chromophore in proximity to the ionization centre so that the protonated and deprotonated species show satisfactory spectra differences^13^. The same approach was also employed in this work to determine the acid-base behaviour of the alkaloids against varying pH and temperature dependence^14^. In this communication, we report the antiplasmodial and antioxidant activities for isocorydine **1** and norisocorydine **2** (Table 1). In addition, the p*K*_*a*_ values of isocorydine **1**, norisocorydine **2** and boldine **3** were also determined (Table 2). All graphs of the pH-absorbance of alkaloids **1**–**3** can be found as supplementary Fig. S2–S4 online.

## Results

**Figure 1:**
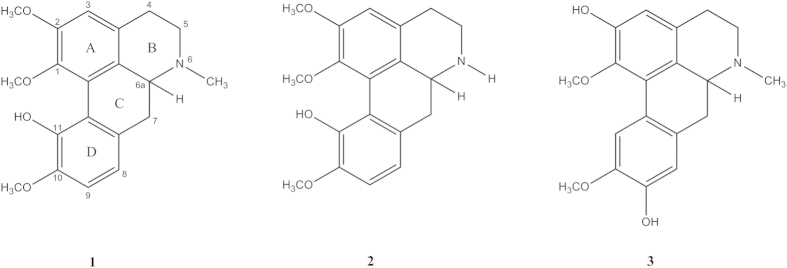
Structures of isocorydine (**1**), norisocorydine (**2**), boldine (**3**).

The chemical investigation contents of the crude dichloromethane extract of *A.corneri* yielded two aporphine alkaloids namely isocorydine **1** and norisocorydine **2**, together with boldine **3** that was isolated from the bark of *D.longipedicellata* (Fig. 1). The structural elucidation was established through several spectroscopic methods; UV, IR, MS, 1D and 2D-NMR (^1^H, ^13^C, DEPT, COSY, HMQC, HMBC) and also by comparison with literature data^15^,^16^. Previously, boldine **3** exhibited potent antiplasmodial activity with IC_50_ 2.60 μM^17^, follow by norisocorydine **2** and isocorydine **1** with IC_50_ 19.8 and 51.3 μM respectively (Table 1). Furthermore, to obtain the full potential of all the alkaloids, three antioxidant assays namely; DPPH, FRAP and iron chelating activities were carried out (Table 1). Norisocorydine **2** and boldine **3** showed free radical DPPH scavenging activity with an IC_50_ of 93.46 μM and 137.13 μM, respectively^17^. All alkaloids showed iron chelating and FRAP activities (Table 1).

**Figure 2:**
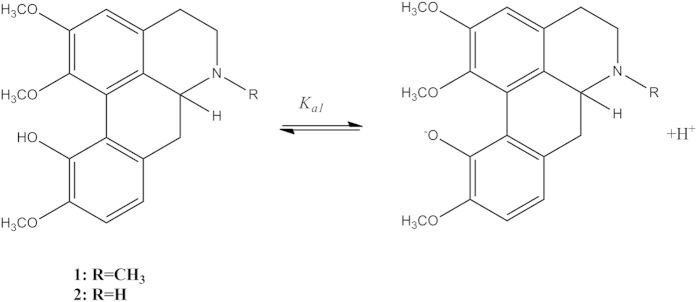
Acid-base equilibria for isocorydine (**1**) norisocorydine (**2**).

**Figure 3:**
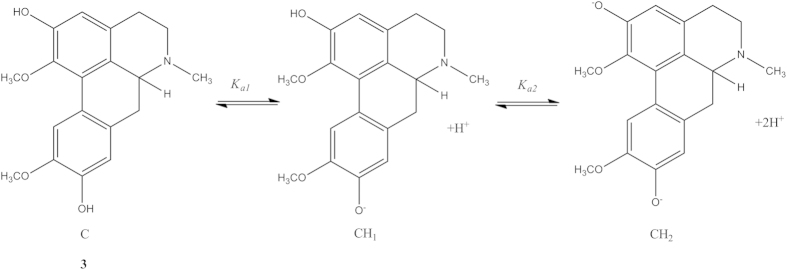
Acid-base equilibria for boldine (**3**).

The general mechanism for the dissociation equilibria of alkaloids **1** to **3** have been proposed as shown in Figs 2 and 3. One acid dissociation constant (*K*_*a*_) was predicted for isocorydine **1** and norisocorydine **2** (Fig. 2), while there were two *K*_*a*_ values for boldine **3** (Fig. 3). In brief, both mechanisms indicated that aporphine protons tend to dissociate from phenolic proton of the phenolic groups.

**Figure 4:**
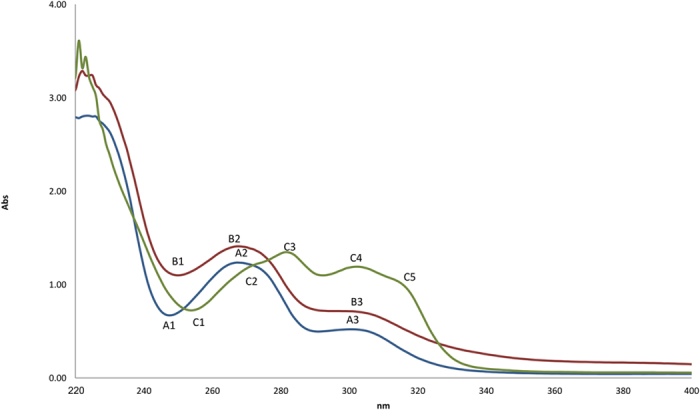
The UV absorption of 2 × 10^−4 ^M isocorydine (**1**) (blue), norisocorydine (**2**) (red), boldine **3** (green) in 2% v/v acetonitrile.

To obtain the potential relationship between the acid-base properties of an alkaloid and its antiplasmodial activity, it is crucial to determine its ionization constant p*K*_*a*_. Figure 4 depicted the UV-vis spectra of alkaloids **1**, **2** and **3** in 2% v/v acetonitrile. The UV-vis spectra of isocorydine **1** and norisocorydine **2**, each with a hydroxyl group at position 11, were similar to each other at all ranges and were characterized by three bands that showed maxima at A1, B1 (246 nm), A2, B2 (270 nm) and A3, B3 (300 nm). In the meantime, boldine **3**, unsubstituted at position 11 was characterized by five bands which showed maxima at C1 (253 nm), C2 (274 nm), C3 (282 nm), C4 (302 nm) and C5 (315 nm), with bands C3, C4 and C5 having equal intensity. In this case, the shapes of the curves and the intensities of the maxima were reliant on the position of the substituents on ring D^18^.

**Figure 5:**
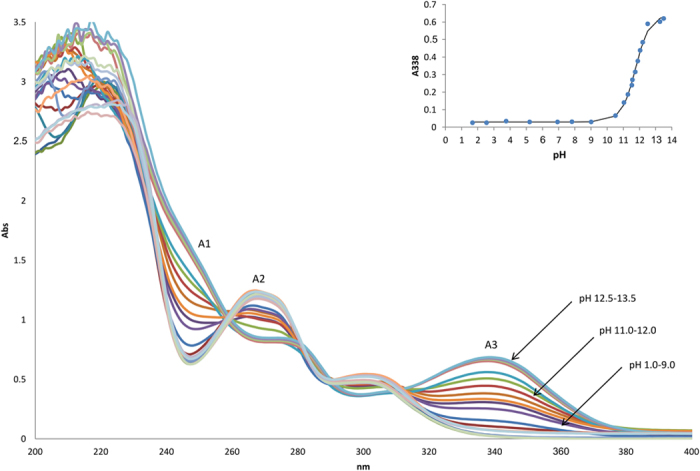
The UV absorption spectra of 2 × 10^−4 ^M isocorydine (**1**) in 2% v/v acetonitrile at pH 1–13.5. Inset shows the pH-dependence of the absorbance at 338 nm.

**Figure 6:**
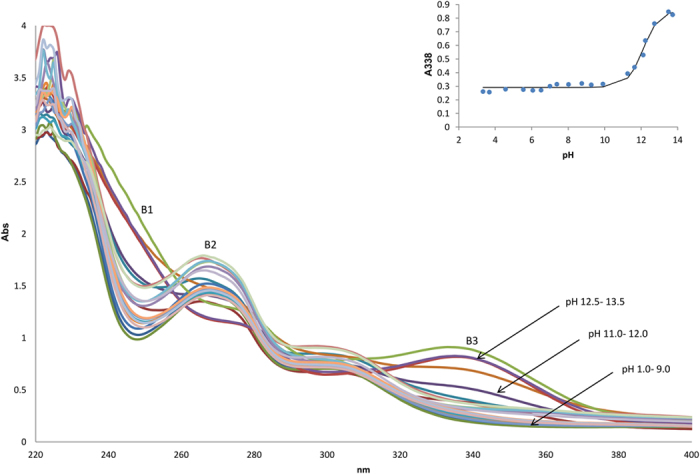
The UV absorption spectra of 2 × 10^−4 ^M norisocorydine (**2**) in 2% v/v acetonitrile at pH 1–13.5. Inset shows the pH-dependence of the absorbance at 338 nm.

**Figure 7:**
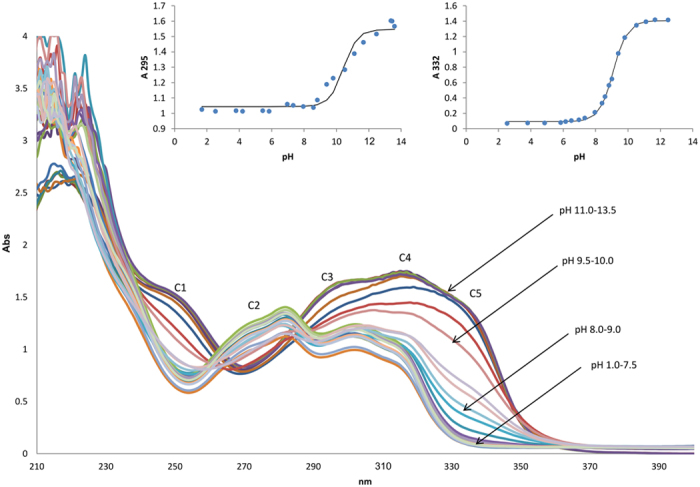
The UV absorption spectra of 2 × 10^−4 ^M boldine (**3**) in 2% v/v acetonitrile at pH 1.0 – 13.5. Inset shows the pH-dependence of the absorbances at 295 nm and 332 nm.

The UV-vis spectra at different pH ranges was monitored in 2% v/v acetonitrile for alkaloids **1**, **2** and **3** to determine the *K*_*a*_ values as shown in Figs 5, 6, 7 respectively. Isocorydine **1** and norisocorydine **2** exhibited no substantial shifts in their wavelengths and changes in their shapes within the pH range of 1.0 to 10.0. Thus, it remained in its neutral form in the acidic medium in which no monocation species was detected^19^. However, spectra differences were observed at 338 nm as a result of changes in the pH from 10.0 to 13.5. As a result of dissociation of the alkaloids in alkaline medium, the UV-vis spectra of isocorydine **1**, norisocorydine **2** undergone substantial bathochromic effect to A3, B3 as shown in Figs 5 and 6. Broad absorption bands between 250 and 350 nm, indicated that the spectra of boldine **3** changes with pH as a result of the presence of two acid-base equilibria in the solution. The two spectra differences were observed at 295 nm and 332 nm. These two bands of boldine **3** also remained in their neutral forms in the acidic medium in which no monocation was detected. Nevertheless, the C3, C4, C5 bands amended hyperchromic and bathochromic effects in the alkaline medium between pH 9.0 to 11.0 as shown in Fig. 7.

Each inset of Figs 5 and 6 revealed the substantial changes of the absorbance within pH 11.0 –12.0 for isocorydine **1** and norisocorydine **2** and inset of Fig. 7 showed two substantial changes of the absorbance at 295 nm between pH 10.0–11.0 and 332 nm between pH 9.0–10.0 for boldine **3**. Thus, this indicated the existence of equilibrium between the ionic species and the neutral species of the alkaloids. An *S*-shaped curve was obtained using the absorbance (Abs)-pH relation for each alkaloid, thus obeying Eq. 1.


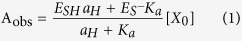


where, A_obs_ = absorbance of substrate, pH = −log *a*_H_ (under dilute conditions, *a*_H_ = [H^+^], *K*_*a*_ = acidity constant, E_SH_ = extinction coefficient of protonated substrate, E_S_^−^ = extinction coefficient of deprotonated substrate and [X_0_] = initial concentration of substrate. The values of p*K*_*a*_ = −log *K*_*a*_, E_SH_ and E_S_^−^ were calculated from equation (1) by the use of nonlinear least squares technique. This equation was actually derived from the equation that shows the relationship of the ionization constant with the Beer Lambert law where the ratio of the ionic to neutral species depends upon the pH at which the solution was optically measured. The calculated values of *K*_*a*_, E_SH_, E_S_^−^ and p*K*_a_ are listed in Table 2. The calculated data associated with low residual errors can be found as supplementary Tables S1–S11.

The calculated p*K*_*a*_ values are given in Table 2 for all of the alkaloids that have been studied at 35 °C. The values for isocorydine **1** (p*K*_*a*_ 11.75) and norisocorydine **2** (p*K*_*a*_ 12.07) were notably similar. This could be expected based on their similar structural features with the only difference in the methylation of *N*-2 in isocorydine **1**. Meanwhile, boldine **3** that has a different value and position of substituent compared to both alkaloids gave much lower value (p*K*_*a*_ 9.16, 10.44). The p*K*_*a*_ values for these three alkaloids can be compared to methoxyphenol that has p*K*_*a*_ values around 9.29–10.50^20^.

These differences could be related to the steric factor since isocorydine **1** and norisocorydine **2** are hydroxyl substituted at position 11, while boldine **3** is substituted at position 2 and 9 instead. Steric effects can influence the p*K*_*a*_ value by distorting the molecular structure from planarity or otherwise disrupting the electronic system of the acid^21^. The intramolecular hydrogen bonding between the 1-OCH_3_ group in ring A and the 11-OH group in ring D resulted in isocorydine **1** and norisocorydine **2** possessing a higher p*K*_*a*_ value as compared to that of boldine **3**. This type of intramolecular hydrogen bonding was not observed in boldine **3** due to the absence of 11-OH group as in skeleton^22^. Boldine **3**, on the other hand exhibit two p*K*_*a*_ values due to the presence of two hydroxyl groups; 2-OH and 9-OH, in its ring A and D respectively. The lower p*K*_*a*_ values of the latter could have resulted from the intersystem crossing of electrons between rings D to A via resonance of the electron donating 9-OH group. As for the 2-OH group, its higher p*K*_*a*_ values maybe due to the absence of the intersystem crossing of electrons between ring A to D via resonance because the 2-OH group in ring A acts as an electron acceptor^23^. The additional p*K*_*a*_ values of boldine **3** at 9.57 and 9.56 belong to the absorbance at 312 and 253 nm respectively (Table 2) may be referred to the mean value of the two ionization constants corresponding to the 2-OH and 9-OH groups.

**Figure 8:**
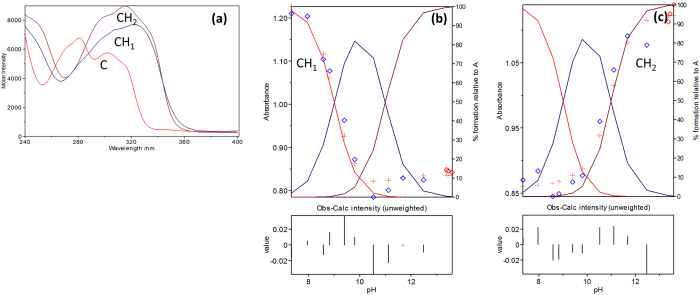
(**a**) Molar absorbance, (**b**) Distribution curve spectra at p*K*_*a*_ 9.11 and (**c**) Distribution curve spectra at p*K*_*a*_ 10.75 of boldine **3**.

The acidity constant, molar absorbance spectra and the distribution diagram of boldine **3** (Fig. 8) were evaluated by the HypSpec programme using absorbance-pH data and proposed chemical model as input (supplementary Fig. S1). The calculated p*K*_*a*_ values with standard deviations from these two programmes (Basica and HypSpec 2014) ascertained that boldine **3** gave two p*K*_*a*_ values (Table 3). Details of calculation methods and computer programs have already been reported^24^. The correlation coefficient for this calculation gave r^2^ = 0.961. The standard deviations associated with the calculated p*K*_*a*_ values revealed very satisfactory data fitting.

In addition, temperature variations also influence the effective mobility of the alkaloids via its degree of ionisation as can be seen in Table 4. Norisocorydine **2** showed that the increase in the temperature will lead to a decrease in its p*K*_*a*_ values. This can be exemplified by the van’t Hoff equations Eqs 2 and 3 where,


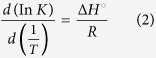



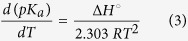


**Figure 9:**
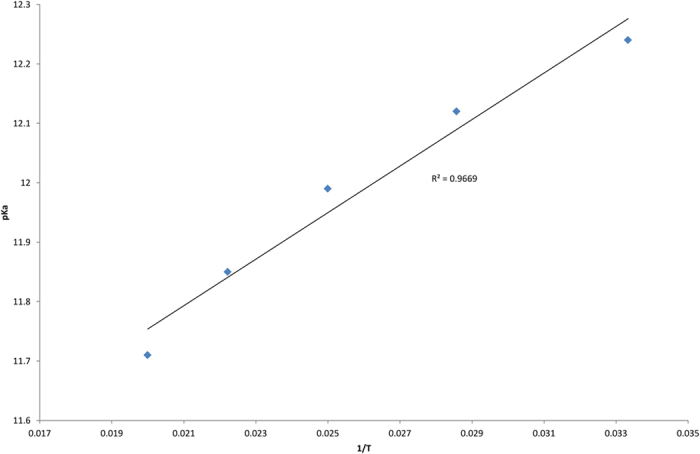
Graph p*K*_*a*_ of 2 × 10^−4 ^M norisocorydine (**2**) at 338 nm in water/acetonitrile, *I* = 0.1 M (NaCl), versus different temperature 30–50 °C.

∆H° is assumed constant and independent of temperature. When the dissociation is exothermic (∆H° < 0), the p*K*_*a*_ will increase with increasing temperature, and when it is endothermic (∆H° > 0), p*K*_*a*_ will decrease. The experimental data are in agreement with the Le Chatelier’s principle which is the addition of heat to a reaction will favour the endothermic direction of a reaction as this reduces the amount of heat produced in the equilibrium system^21^,^25^,^26^. As a conclusion to this principle, a graph of p*K*_*a*_ versus (1/T) was plotted for norisocorydine **2** at 338 nm. This gave a straight line with a regression value of 0.97, indicating that the acid dissociation was temperature dependant (Fig. 9).

## Discussion

*Plasmodium* parasite invades the host hemoglobin as a source of amino acids for its own survival. During this process, the host hemoglobin is destroyed and liberates free electron and free heme. Free electron is formed from the oxidation of iron bound in haemoglobin Fe^2+^ to Fe^3+^. The release of free electrons will produce free radicals called reactive oxygen species (ROS). Generation of ROS is associated with oxidative stress and it is highly toxic to the host cell that will lead to hemolysis or cell damage^27^. Therefore, the presence of alkaloids **1**–**3** that possess antioxidant activity will prevent the oxidative damage to the hosts due to the ability of the hydroxyl groups which can chelate iron and donate electrons to free radicals in the ROS^28^. In addition, free heme (Fe atom) will also be released during haemoglobin destruction. This free heme eventually will convert to hemazoin. Hemazoin is important for the survival of *Plasmodium* parasites. The alkaloids are able to bind to the toxic free heme and thus prevent the formation of hemazoin^27^,^29^,^30^,^31^.

It is well known that acidic food vacuole in *Plasmodium* is a special organelle for the digestion of the host haemoglobin, storage site for hemazoin and the site of action of many antimalarial drugs and this food vacuole has a pH around 5.5^32^. Therefore, it is important to find molecules that can cross the erythrocyte and parasite membranes to neutralize the parasite acidic food vacuole. Phenolic aporphine alkaloids are amphoteric and more stable in acidic rather than in alkaline medium. In acidic and physiological pH medium, the aporphines exist as non-ionized molecules and its basic nitrogen will be protonated to accumulate within the acidic parasite food vacuole via pH trapping. Once protonated, they are trapped in the food vacuole resulting in the increased drug accumulation at the target site, and hence enhancing antiplasmodial activity^33^. Usually the majority of important drugs belong to the class of weak acids or weak bases as they can be present in solution as both the non-ionized and ionized species. As an example for alkaloids, the antimalarial drug chloroquine has p*K*_*a*_ values around 8.35 and 10.4^34^. Therefore, the transmembrane distribution of a weak electrolyte is influenced by its p*K*_*a*_ and the pH gradient across the membrane. The p*K*_*a*_ is the pH at which half of the drug concentration (weak acid or base electrolyte) is in its ionized form^35^,^36^.

The results indicated a positive correlation between the *in vitro* antiplasmodial and antioxidant activities for alkaloids **1**–**3**. Among all the alkaloids, boldine **3** showed p*K*_*a*_ around 9.16 and 10.44 that was most comparable to chloroquine p*K*_*a*_ (8.35, 10.4). Based on pharmacodynamics and pharmacokinetic characteristics, it is noteworthy that slightly basic properties of the aforementioned alkaloids, along with their antioxidant activities, are advantageous in improving the suppression of malaria infection that cause less damage to the host.

## Experimental

### Plant Material

The leaves of *Alseodaphne corneri* Kosterm. was collected from Hutan Simpan Kenderong, Gerik, Perak, Malaysia. The bark of *Dehaasia longipedicellata* (Ridl.) was collected from Sungai Tekam Reserve Forest, Jerantut, Pahang, Malaysia. The plant specimens were identified by Mr. Teo Leong Eng and Mr. Din Mat Nor. Voucher specimens (KL5641 and KL 5634) were deposited at the Herbarium of the Department of Chemistry, University of Malaya, Kuala Lumpur, Malaysia.

### Extraction and Separation

Plant extraction was carried out by cold percolation. Dried grounded leaves of *A.corneri* (1.5 kg) and the bark of *D.longipedicellata* (2.0 kg) were initially defatted with hexane (17L) for three days at room temperature. Then, the hexane extract was filtered and dried on the rotary evaporator. The residues were dried and then later moistened with 25% ammonia solution and left for 2 hours. They were then re-extracted with dichloromethane (CH_2_Cl_2_) for three days and then CH_2_Cl_2_ extract was dried using a rotary evaporator to a volume of approximately 500 ml. Then, it was re-extracted with 5% of hydrochloric acid and monitored with Mayer’s reagent test. The hydrochloric solution obtained was basified with NH_3_ solution to a pH of 11 followed by extraction with CH_2_Cl_2_ again. Afterward, CH_2_Cl_2_ was washed with distilled water and dried with sodium sulphate anhydrous to remove remaining aqueous solution. Finally, the organic solution was concentrated using rotary evaporator to give CH_2_Cl_2_ of alkaloid from *A.corneri* (4.67 g) and *D.longipedicellata* (10.5 g). The CH_2_Cl_2_ crudes were subjected to column chromatography using silica gel (0.04–0.063 mm; 6 × 65 cm) as the stationary phase using mixtures of (dichloromethane: methanol) as the eluting solvent (100:0, 99:1, 97:3, 96:4, and 90:10) to obtain eight fractions (A1-A8) for *A.corneri* and seven fraction (D1-D7) for *D.longipedicellata.* Purification of A2 and A3 by Preparative Thin Layer Chromatography (PTLC) led to the isolation of alkaloids **1** [CH_2_Cl_2_:MeOH with 97:3; v/v] and **2** [CH_2_Cl_2_:MeOH with 97:3; v/v] saturated with NH_4_OH; Dragendroff reagent. Alkaloid **3** [CH_2_Cl_2_: MeOH with 96:4; v/v] saturated with NH_4_OH; was obtain by preparative-TLC from fraction D4.

### Antiplasmodial Assay

The antiplasmodial activity of **1** and **2** was evaluated against the chloroquine-resistant FcB1 strain of *P. falciparum*. *P. falciparum* was maintained continuously *in vitro* in human erythrocytes according to Trager and Jensen^37^. The antiplasmodial activity was determined according to Desjardin^38^. Alkaloids were dissolved in dimethylsulfoxide (DMSO) and tested at a concentration of 10 μg/ml. Alkaloids showing significant inhibition rates were submitted to serial dilutions with culture medium before being added to asynchronous parasite cultures (1% parasitemia and 1% final hematocrite) in 96-well microplates for 24 h at 37 °C. A concentration of 0.5 μCi of [3H] hypoxanthine was then added to each well, and parasites were maintained for an additional 24 h. The growth inhibition for each compound concentration was determined by comparing the radioactivity incorporated in the treated culture with that in the control culture maintained on the same plate. The concentrations causing 50% inhibition of parasite growth (IC_50_) were calculated from the drug concentration-response curves are presented in Table 1.

## Antioxidant Assay

### DPPH assay

The DPPH scavenging activity of purified alkaloids were tested based on the method previously published^39^. Briefly, 40 μL of purified alkaloids at different concentrations were mixed with 200 μL of 50 μM DPPH solution in MeOH. The mixture was immediately shaken and incubated for 15 min in the dark at room temperature. The decrease in absorbance was measured at 517 nm with a microplate reader (Tecan Sunrise, Austria). BHA was used as a standard and the control was MeOH. The percentage of inhibition activity of the alkaloids was calculated (*n* = 3) and results are presented in Table 1.

### Metal chelating activity assay

The ferrous ion chelating activity of the purified alkaloids was determined according to the previously published^39^ by measuring the formation of the Fe^2+^- ferrozine complex in the reaction mixture. Briefly, 100 μL of purified alkaloids or standards (6.25–100 μg/mL) were mixed with 120 μL distilled water and 10 μL FeCl_2_ (2 mM) in a 96-well microplate and the absorbance was read as blank. Then, 20 μL of Ferrozine (5 mM) was added to the mixture to initiate the reaction. The reaction mixture was incubated at room temperature for 20 min and the absorbance at 562 nm was measured. The results are presented in Table 1.

### FRAP

The FRAP activities of alkaloids were measured according to the previously published method^39^. Twenty microliters of alkaloids in MeOH were mixed with, 30 μL of water, 200 μL of daily prepared FRAP reagent [1 volume of 10 mM TPTZ in 40 mM HCl, 1 volume of 20 mM FeCl_3_, and 10 volumes of 0.3 M acetate buffer (pH 3.6)] in 96-well microplate. After 8 min of incubation time, the formation of the TPTZ-Fe^2+^ complex in the presence of antioxidant compounds was detected at 595 nm with a microplate reader (Tecan Sunrise, Austria). Ferrous sulfate (FeSO_4_) solution (50 to 1000 μM) was used for standard calibration curve. The FRAP value of alkaloids which is required for the reduction of Fe^3+^ to Fe^2+^ was calculated (*n* = 3) and results are reported as μM Fe^2+^ in Table 1.

### Reagents and materials

Isocorydine **1** and norisocorydine **2** were isolated from the leaves of *Alseodaphne corneri*, while boldine **3** was isolated from the bark of *D.*longipedicellata. All other chemicals were obtained from Fluka, Merck and Sigma, as reagent grade materials. Deionized water was used in the preparation of the buffer solutions. The alkaloids are not appreciably soluble in water; therefore the stock solutions of the alkaloids were prepared in an appropriate volume of distilled acetonitrile. The experiment was performed in a mixture of acetonitrile-water (2% v/v acetonitrile) HCl and NaOH of various concentrations were used to cover the strongly acidic and basic regions. Buffer solutions of glycine, formate, acetate, MES, MOPS, HEPES and TRIS were used to guard the pH ranges from 3.0 to 11.0. The preparation of these buffers followed the standard method published by Perrin and Dempsey^40^,^41^.

### Instrumentation

Spectra were recorded using the following instruments; UV, Shimadzu UV-250, UV-Visible spectrometer; IR, Perkin Elmer 1600; NMR, AVN BRUKER with TMS as internal standard and CDCl_3_ as the solvent to obtain the 400 MHz proton and 100 MHz carbon spectra. Mass spectra were obtained using on Agilent technologies 6530 Accurate-Mass Q-TOF liquid chromatography/Mass spectrometry (LCMS), with ZORBAX Eclipse XDB-C18 Rapid Resolution HT 4.6 mm i.d × 50 mm × 1.8 μm column. All solvents, except those used for bulk extraction were AR grade. Column chromatography separations were conducted by using Merck silica gel 60 (230–400 mesh) and silica gel 60 F254 for thin layer chromatography (TLC) monitoring. Glass and aluminium supported silica gel 60 F254 plates were used for TLC. TLC spots were visualized under UV light (254 and 365 nm) followed by spraying with Dragendroff’s reagent for alkaloid detection. Studies for determination of acid dissociation constant of the alkaloids were carried out by using a Shimadzu 1650 PC UV/vis double beam Spectrophotometer equipped with multicell compartment and peltier-controlled temperature. Quartz cells with 1 cm path length were used both as reference and blank sample. The pH of the solutions was measured by Mettler Toledo Model S40 digital pH meter with an accuracy of ±0.01 units. The meter was equipped with a combined pH electrode with ATC temperature detector filled with a solution of 3 M KCl and was standardized using standard aqueous buffers (pH 4.01, 7.00 and 9.21 at 35 °C).

### Spectrophotometric measurements

The experimental reaction mixtures were prepared by diluting the appropriate amount of the stock solutions of alkaloids **1**–**3** in a pre-prepared buffer solution to give the compound concentration of 2.0 × 10^−4 ^M. The ionic strength was maintained at 0.10 M with NaCl at 35 °C. Deionized water and pure acetonitrile were used to prepare the solutions of 2% v/v acetonitrile. The UV/vis spectra were monitored from 190 nm–400 nm for each compound. The reference cell contained deionized water or acetonitrile, for measurements done in 2% v/v acetonitrile, respectively. The temperature of the sample was maintained in a thermostated waterbath. While the spectrum was running, the pH of the sample was measured at an appropriate temperature using a pH electrode attached to a digital pH meter equipped with an automatic temperature probe. The electrode was calibrated at the same temperature as the sample using standard buffers of known pH at the sample temperature.

### Determination of acidity constants

The acidity constants of the alkaloids were determined from their spectra behaviour in buffer solutions covering a pH range from 1 to 14 at selected wavelengths. Absorbance at a specific wavelength was recorded and the acidity constants (p*K*_*a*_, *K*_*a*_) were calculated based on (equation 1) using Basica (see Supplementary Data 1)^42^ and HypSpec programmes^24^.

## Conclusion

The UV-vis spectra of isocorydine **1**, norisocorydine **2** have similar features when compared to boldine **3.** The UV-vis spectra of all the alkaloids remained unchanged in acidic condition; however substantial bathochromic shifts were observed due to the deprotonation of the phenolic protons in basic condition. The p*K*_*a*_ values of isocorydine **1** and norisocorydine **2** were 11.75 and 12.07, respectively. Meanwhile, boldine **3** gave two p*K*_*a*_ values of 9.16, 10.44 from Basica and 9.12, 10.75 from HypSpec programs. Therefore, the p*K*_*a*_ values are substantially dependent on the position of the substituents. Moreover, all alkaloids showed p*K*_*a*_ values above the physiological pH; thereby all of them will not ionize at physiological pH, thus permitting the basic nitrogen to be protonated and accumulated within the acidic food vacuole of *Plasmodium* via pH trapping. Subsequently, acidic food vacuoles that have been neutralized by alkaloids would result in the enhancement antiplasmodial activity. Interestingly, these alkaloids also possessed antioxidant activities that will prevent oxidative damage to the host by binding to the ferum by preventing the formation of hemazoin. Among all of the alkaloids, boldine **3** showed comparable antiplasmodial, antioxidant and p*K*_*a*_ values to chloroquine, an example of antimalarial drugs. In summary, one may suggest that the use of alkaloids having antioxidant properties that contains slightly basic functionalities may be far more effective antimalarial drugs that cause less damage to the host.

## Additional Information

**How to cite this article**: Zahari, A. *et al.* Ultraviolet-visible study on acid-base equilibria of aporphine alkaloids with antiplasmodial and antioxidant activities from *Alseodaphne corneri* and *Dehaasia longipedicellata*. *Sci. Rep.* **6**, 21517; doi: 10.1038/srep21517 (2016).

## Supplementary Material

Supplementary Information

## Figures and Tables

**Table 1:** Antiplasmodial and antioxidant activities of isolated alkaloids from *Alseodaphne corneri*.

Alkaloids	IC_50_ FcB1 (μM)	IC_50_ DPPH Activity (μM)	IC_50_ Ferum Metal Chelating Activity (μM)	FRAP (μM Fe^2+^)
isocorydine **1**	51.30 ± 0.61	229.85 ± 7.51	88.06 ± 6.04	0.79 ± 0.04
norisocorydine **2**	19.80 ± 0.26	93.46 ± 7.85	114.40 ± 3.92	1.05 ± 0.13
Chloroquine	0.090 ± 0.03			
BHA (Standard)		26.47 ± 4.16		
EDTA-Na (Standard)			20.01 ± 5.32	

**Table 2:** Values of ionization constant for alkaloids 1–3 (2 × 10^−4 ^M) in 2% v/v acetonitrile, *I* = 0.1 M (NaCl), at 35 °C.

Alkaloids	λ/nm	*K*_*a*_^a^ (M)	p*K*_*a*_^b^	10^−2^ E_SH_^a^ (M^−1^ cm^−1^)	10^−2^ E_S_^−^a (M^−1^ cm^−1^)
isocorydine **1**	338	(1.77 ± 0.1) × 10^−12^	11.75	2.9 ± 0.5^c^	64.0 ± 0.9^c^
	270	(2.24 ± 0.3) × 10^−12^	11.65	119.1 ± 0.6	78.4 ± 1.1
	246	(1.75 ± 0.02) × 10^−12^	11.76	63.9 ± 0.9	161.9 ± 1.8
norisocorydine **2**	338	(7.72 ± 1.1) × 10^−13^	12.11	29.1 ± 0.7	85.5 ± 1.9
	270	(7.63 ± 5.2) × 10^−13^	12.12	141.0 ± 2.5	99.5 ± 5.2
	246	(1.02 ± 0.5) × 10^−12^	11.99	115.2 ± 2.7	193.1 ± 6.3
boldine **3**	332	(7.61 ± 0.3) × 10^−10^	9.12	11.5 ± 0.8	141.0 ± 0.8
	312	(2.68 ± 0.5) × 10^−10^	9.57	96.2 ± 1.4	116.7 ± 1.7
	295	(3.59 ± 1.2) × 10^−11^	10.44	104.4 ± 1.5	154.6 ± 2.2
	274	(6.19 ± 1.5) × 10^−10^	9.21	113.6 ± 1.1	77.4 ± 1.6
	253	(2.74 ± 0.5) × 10^−10^	9.56	64.5 ± 1.2	132.0 ± 2.1

^a^calculated from equation (1).

^b^calculated from p*K*_*a*_ = (−log *K*_*a*_).

^c^errors limits are standard deviations.

**Table 3:** p*K*_*a*_ values of alkaloids 1–3 at 35 °C obtained using Basica and HypSpec 2014.

Alkaloids	p*K*_*a*1_	p*K*_*a*2_	Software
Isocorydine **1**	11.75 ± 0.06	–	Basica
Norisocorydine **2**	12.07 ± 0.07	–	Basica
Boldine **3**	9.16 ± 0.06	10.44 ± 0.01	Basica
	9.12 ± 0.02	10.75 ± 0.01	HypSpec

**Table 4:** Values of ionization constant for norisocorydine 2 (2 × 10^−4 ^M) in 2% v/v acetonitrile, *I* = 0.1 M (NaCl), at 35–50 °C.

T/°C	*K*_*a*_^a^ (M)	p*K*_*a*_^b^	10^−2^ E_SH_^a^ (M^−1^ cm^−1^)	10^−2^ E_S_^−^a (M^−1^ cm^−1^)
**30**	(5.80 ± 0.86) × 10^−13^	12.24	23.2 ± 0.7^c^	80.0 ± 1.6
**35**	(7.72 ± 1.08) × 10^−13^	12.07	29.1 ± 0.7	85.5 ± 1.9
**40**	(10.14 ± 1.45) × 10^−13^	11.99	22.9 ± 0.7	81.5 ± 1.8
**45**	(14.41 ± 2.11) × 10^−13^	11.84	22.8 ± 0.7	82.4 ± 2.1
**50**	(19.43 ± 3.09) × 10^−13^	11.71	22.8 ± 0.8	81.5 ± 2.2

^a^calculated from equation (1).

^b^calculated p*K*_*a*_ = (−log *K*_*a*_).

^c^errors limits are standard deviations.
