# Supplementary material for: Ultraviolet-visible study on acid-base equilibria of aporphine alkaloids with antiplasmodial and antioxidant activities from Alseodaphne corneri and Dehaasia longipedicellata
Source: Sci Rep. 2016 Feb 22;6:21517. doi: 10.1038/srep21517 (PMC4761911; doi:10.1038/srep21517)
Supplement: Supplementary Information [file srep21517-s1.pdf]

Ultraviolet-visible study on acid-base equilibria of aporphine alkaloids with antiplasmodial and antioxidant activities from *Alseodaphne corneri* and *Dehaasia longipedicellata*.

Azeana Zahari [1], Abdulwali Ablat [2], Noridayu Omer [1], Mohd Azlan Nafiah[3], Yasodha Sivasothy [1], Jamaludin Mohamad [2], Mohammad Niyaz Khan[1], Khalijah Awang [1,] *

[1] Department of Chemistry, Faculty of Science, University of Malaya, 50603 Kuala Lumpur, Malaysia.

[2] Institute of Biological Sciences, Faculty of Science, University of Malaya, 50603 Kuala Lumpur, Malaysia.

[3] Chemistry Department, Faculty of Science and Mathematics, University of Pendidikan Sultan Idris, 35900 Tanjung Malim, Perak, Malaysia.

boldine model

Maximum number of iterations 25 Weights - absolute

Formula	Log Beta	A	H	
AH	11.0	1	1	refine
AH_2	20.0	1	2	refine

Supplementary Figure S1. Proposed chemical model of Boldine **3**.

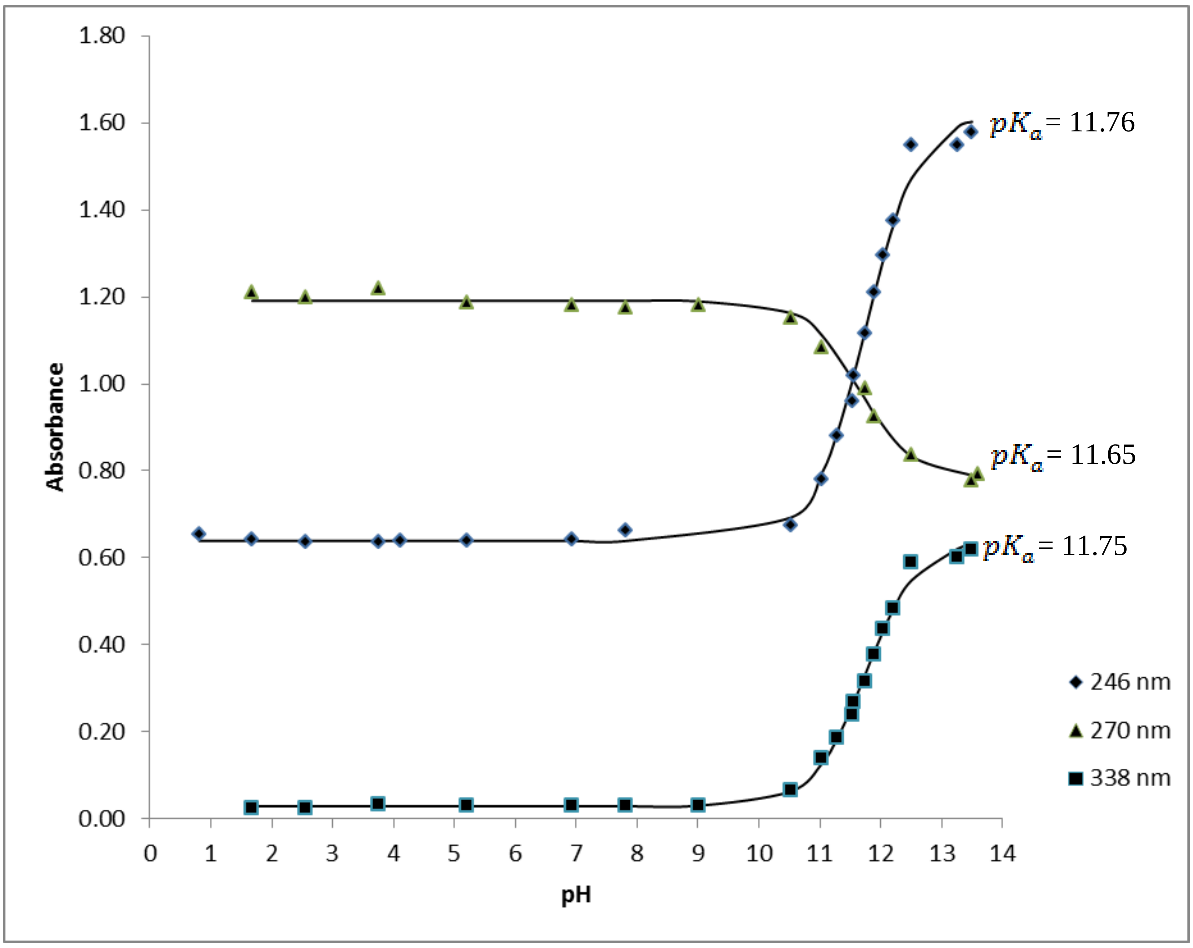

Supplementary Figure S2. pH-Absorbance curves of Isocorydine **1** in 2% v/v acetonitrile at 246 nm, 270 nm, and 338 nm.

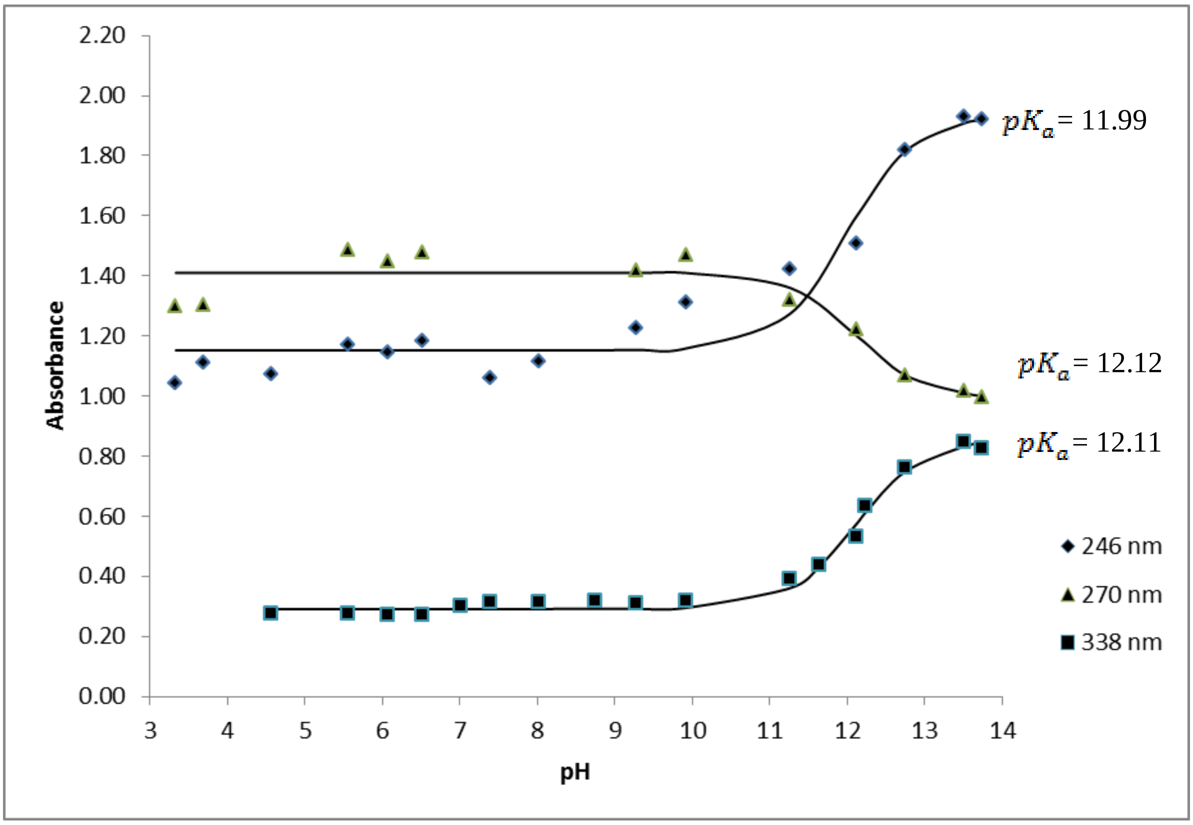

Supplementary Figure S3. pH-Absorbance curves of Norisocorydine **2** in 2% v/v acetonitrile at 246 nm, 270 nm, and 338 nm.

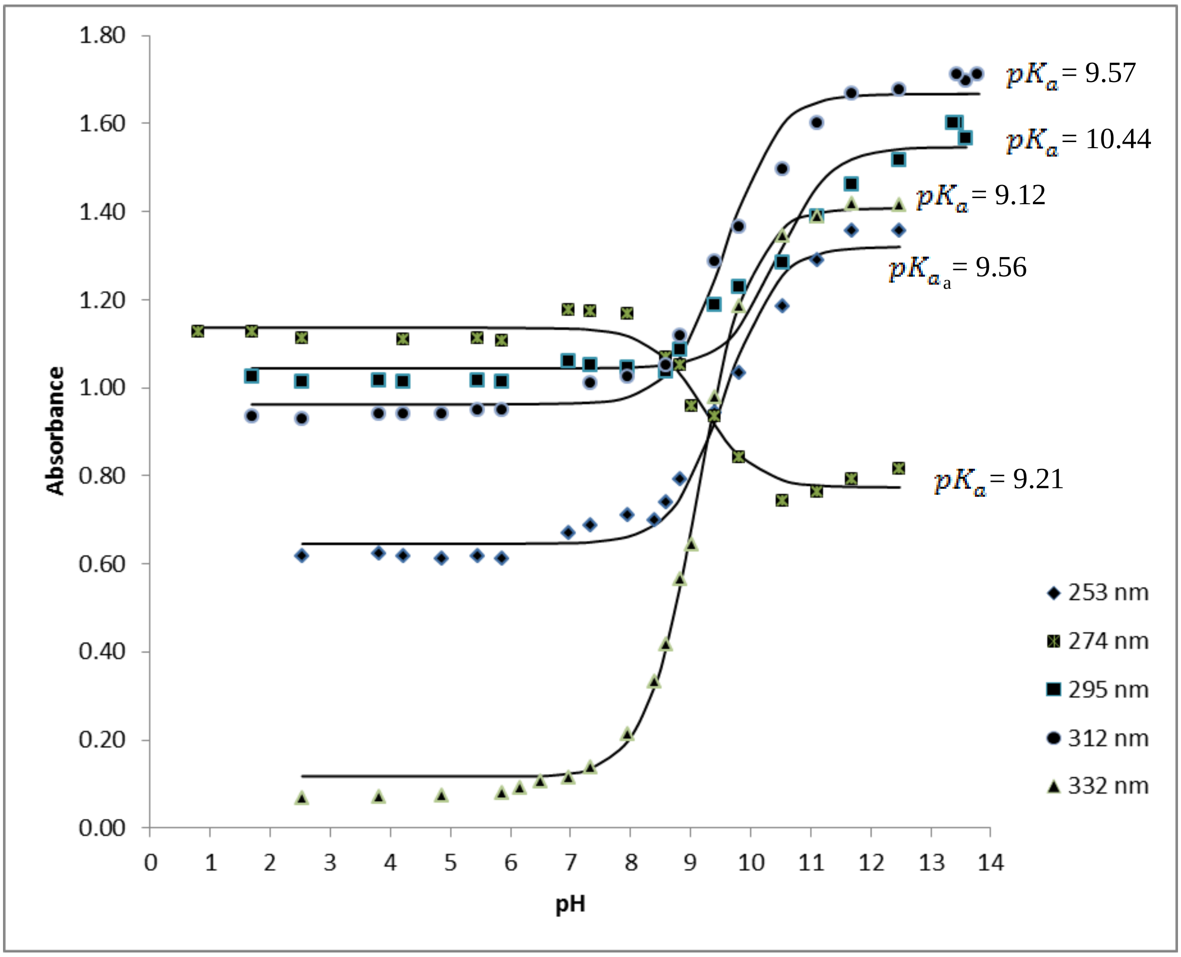

Supplementary Figure S4. pH-Absorbance curves of Boldine **3** in 2% v/v acetonitrile at 253 nm, 274 nm, 295 nm, 312 nm and 332 nm.

Supplementary Table S1 The values of observed absorbance (A_{obs}) at 338 nm as a function of pH for ionization of Isocorydine **1** in 2% v/v acetonitrile

pH	A_{obs}	A_{calc}	%RE
13.50	0.620	0.629	-1.43
13.27	0.602	0.622	-3.25
12.51	0.589	0.549	6.82
12.20	0.484	0.479	0.99
12.04	0.438	0.432	1.35
11.89	0.377	0.383	-1.47
11.74	0.315	0.330	-4.85
11.56	0.270	0.268	0.68
11.53	0.240	0.258	-7.58
11.28	0.187	0.183	1.95
11.04	0.140	0.129	8.10
10.52	0.066	0.063	4.09
9.01	0.030	0.031	-2.13
7.82	0.031	0.030	4.49
6.93	0.030	0.030	1.51
5.21	0.030	0.030	1.54
3.75	0.034	0.030	13.13
2.55	0.025	0.030	-18.15
1.68	0.025	0.030	-18.15

$K_a =$ $(1.769 \pm 0.098) \times 10^{-12}$

$10^{-2}\ E_{SH} =$ 2.95 ± 0.47

$10^{-2}\ E_{S^-} =$ 63.95 ± 0.91

Supplementary Table S2 The values of observed absorbance (A_{obs}) at 270 nm as a function of pH for ionization of Isocorydine **1** in 2% v/v acetonitrile

pH	A_{obs}	A_{calc}	%RE
13.6	0.794	0.789	0.68
13.5	0.777	0.790	-1.64
12.51	0.838	0.833	0.54
11.89	0.926	0.933	-0.73
11.74	0.991	0.967	2.47
11.04	1.084	1.111	-2.46
10.52	1.153	1.163	-0.84
9.01	1.181	1.190	-0.74
7.82	1.175	1.191	-1.33
6.93	1.181	1.191	-0.82
5.21	1.187	1.191	-0.31
3.75	1.220	1.191	2.41
2.55	1.200	1.191	0.78
1.68	1.211	1.191	1.68

$K_a =$ $(2.24 \pm 0.3) \times 10^{-12}$

$10^{-2}\ E_{SH} =$ 119.07 ± 0.63

$10^{-2}\ E_{S^-} =$ 78.4 ± 1.1

Supplementary Table S3 The values of observed absorbance (A_{obs}) at 246 nm as a function of pH for ionization of Isocorydine **1** in 2% v/v acetonitrile

pH	A_{obs}	A_{calc}	%RE
13.50	1.578	1.602	-1.55
13.27	1.548	1.591	-2.76
12.51	1.550	1.473	4.99
12.20	1.376	1.360	1.17
12.04	1.295	1.284	0.86
11.89	1.211	1.204	0.58
11.74	1.117	1.120	-0.26
11.56	1.019	1.020	-0.11
11.53	0.959	1.004	-4.71
11.28	0.880	0.884	-0.49
11.04	0.780	0.797	-2.17
10.52	0.675	0.693	-2.63
7.82	0.663	0.639	3.60
6.93	0.643	0.639	0.62
5.21	0.639	0.639	-0.002
4.12	0.639	0.639	-0.002
3.75	0.638	0.639	-0.16
2.55	0.636	0.639	-0.47
1.68	0.644	0.639	0.77
0.81	0.654	0.639	2.29

$K_a = (1.750 \pm 0.019) \times 10^{-12}$

$10^{-2}\ E_{SH} = 63.90 \pm 0.89$

$10^{-2}\ E_{S^-} = 161.99 \pm 1.81$

Supplementary Table S4 The values of observed absorbance (A_{obs}) at 338 nm as a function of pH for ionization of Norisocorydine **2** in 2% v/v acetonitrile

pH	A_{obs}	A_{calc}	%RE
13.73	0.824	0.8415	-2.13
13.51	0.847	0.8331	1.63
12.74	0.760	0.7473	1.67
12.24	0.634	0.6141	3.14
12.12	0.529	0.5754	-8.77
11.64	0.439	0.4332	1.32
11.26	0.392	0.3606	8.01
9.91	0.316	0.2947	6.74
9.27	0.310	0.2920	5.81
8.75	0.320	0.2914	8.93
8.02	0.312	0.2912	6.66
7.39	0.314	0.2912	7.27
7.01	0.300	0.2912	2.94
6.52	0.270	0.2912	-7.84
6.07	0.269	0.2912	-8.24
5.55	0.275	0.2912	-5.88
4.57	0.277	0.2912	-5.11
3.68	0.256	0.2912	-13.74
3.33	0.260	0.2912	-11.99

$K_a = (7.72 \pm 1.08) \times 10^{-13}$

$10^{-2}\ E_{SH} = 29.11 \pm 0.72$

$10^{-2}\ E_{S^-} = 85.5 \pm 1.88$

Supplementary Table S5 The values of observed absorbance (A_{obs}) at 270 nm as a function of pH for ionization of Norisocorydine **2** in 2% v/v acetonitrile

**pH**	$\mathbf{A_{obs}}$	$\mathbf{A_{calc}}$	**%RE**
13.73	0.998	1.000	-0.20
13.51	1.017	1.010	0.69
12.74	1.069	1.070	-0.09
12.12	1.221	1.200	1.72
11.26	1.318	1.360	-3.19
9.91	1.469	1.410	4.02
9.27	1.418	1.410	0.56
6.52	1.477	1.410	4.54
6.07	1.448	1.410	2.62
5.55	1.485	1.410	5.05
3.68	1.301	1.410	-8.38
3.33	1.297	1.410	-8.71

$K_a = (7.63 \pm 5.22) \times 10^{-13}$

$10^{-2}\ E_{SH} = 141 \pm 2.52$

$10^{-2}\ E_{S-} = 99.46 \pm 5.18$

Supplementary Table S6 The values of observed absorbance (A_{obs}) at 246 nm as a function of pH for ionization of Norisocorydine **2** in 2% v/v acetonitrile

**pH**	$\mathbf{A_{obs}}$	$\mathbf{A_{calc}}$	**%RE**
13.73	1.922	1.917	0.24
13.51	1.928	1.909	1.01
12.74	1.819	1.814	0.29
12.12	1.508	1.600	-6.08
11.26	1.423	1.274	10.45
9.91	1.313	1.158	11.78
9.27	1.228	1.153	6.08
8.02	1.117	1.152	-3.13
7.39	1.061	1.152	-8.56
6.52	1.182	1.152	2.55
6.07	1.144	1.152	-0.69
5.55	1.170	1.152	1.55
4.57	1.072	1.152	-7.45
3.68	1.109	1.152	-3.86
3.33	1.044	1.152	-10.33

$K_a = (1.02 \pm 0.47) \times 10^{-12}$

$10^{-2}\ E_{SH} = 115.18 \pm 2.72$

$10^{-2}\ E_{S-} = 193.13 \pm 6.26$

Supplementary Table S7 The values of observed absorbance (A_{obs}) at 332 nm as a function of pH for ionization of Boldine **3** in 2% v/v acetonitrile

**pH**	$\mathbf{A_{obs}}$	$\mathbf{A_{calc}}$	**%RE**
12.48	1.414	1.408	0.45
11.68	1.417	1.405	0.87
11.11	1.390	1.395	-0.37
10.53	1.345	1.360	-1.11
9.80	1.184	1.185	-0.10
9.40	0.978	0.964	1.45
9.01	0.645	0.681	-5.56
8.83	0.564	0.554	1.78
8.58	0.417	0.405	2.92
8.40	0.331	0.322	2.68
7.96	0.213	0.198	6.81
7.34	0.137	0.136	0.92
6.97	0.114	0.124	-8.49
6.50	0.105	0.118	-12.06

$K_a =$ (7.61 ± 0.27) x 10^{-10}

$10^{-2}\ E_{SH} =$ 11.5 ± 0.8

$10^{-2}\ E_{S-} =$ 141 ± 0.8

Supplementary Table S8 The values of observed absorbance (A_{obs}) at 312 nm as a function of pH for ionization of Boldine **3** in 2% v/v acetonitrile

**pH**	A_{obs}	A_{calc}	**%RE**
13.59	1.696	1.667	1.69
13.44	1.712	1.667	2.61
13.77	1.713	1.667	2.67
12.48	1.676	1.666	0.57
11.68	1.668	1.662	0.37
11.11	1.601	1.648	-2.91
10.53	1.495	1.597	-6.86
9.80	1.366	1.406	-2.89
9.40	1.288	1.246	3.25
8.83	1.118	1.070	4.25
8.58	1.051	1.027	2.24
7.96	1.025	0.979	4.49
7.34	1.011	0.966	4.43
5.85	0.950	0.962	-1.29
5.45	0.949	0.962	-1.39
4.85	0.940	0.962	-2.35
4.21	0.940	0.962	-2.35
3.80	0.942	0.962	-2.14
2.53	0.930	0.962	-3.45
1.69	0.934	0.962	-3.01

$K_a = (2.68 \pm 0.5) \times 10^{-10}$

$10^{-2}\, E_{SH} = 96.2 \pm 1.4$

$10^{-2}\, E_{S^-} = 166.7 \pm 1.7$

Supplementary Table S9 The values of observed absorbance (A_{obs}) at 295 nm as a function of pH for ionization of Boldine **3** in 2% v/v acetonitrile

pH	A_{obs}	A_{calc}	%RE
13.59	1.566	1.546	1.27
13.44	1.600	1.546	3.38
13.37	1.602	1.546	3.50
12.48	1.516	1.542	-1.71
11.68	1.462	1.519	-3.89
11.11	1.388	1.457	-4.98
10.53	1.284	1.320	-2.78
9.80	1.228	1.137	7.43
9.40	1.188	1.086	8.62
8.83	1.086	1.056	2.76
8.58	1.037	1.051	-1.34
7.96	1.044	1.046	-0.17
7.34	1.051	1.045	0.62
6.97	1.059	1.044	1.39
5.85	1.012	1.044	-3.17
5.45	1.016	1.044	-2.77
4.21	1.012	1.044	-3.17
3.80	1.016	1.044	-2.77
2.53	1.013	1.044	-3.07
1.69	1.024	1.044	-1.96

$K_a = (3.59 \pm 1.2) \times 10^{-11}$

$10^{-2}\ E_{SH} = 104.4 \pm 1.5$

$10^{-2}\ E_{S-} = 154.6 \pm 2.2$

Supplementary Table S10 The values of observed absorbance (A_{obs}) at 274 nm as a function of pH for ionization of Boldine **3** in 2% v/v acetonitrile

**pH**	$\mathbf{A_{obs}}$	$\mathbf{A_{calc}}$	**%RE**
12.48	0.815	0.774	5.04
11.68	0.793	0.775	2.28
11.11	0.763	0.778	-2.00
10.53	0.743	0.790	-6.36
9.80	0.841	0.848	-0.79
9.40	0.934	0.916	1.97
9.01	0.958	0.996	-3.93
8.83	1.050	1.029	1.97
8.58	1.068	1.067	0.07
7.96	1.168	1.117	4.37
7.34	1.174	1.131	3.62
6.97	1.175	1.134	3.47
5.85	1.107	1.136	-2.63
5.45	1.111	1.136	-2.27
4.21	1.108	1.136	-2.55
2.53	1.113	1.136	-2.09
1.69	1.126	1.136	-0.92
0.80	1.126	1.136	-0.92

$K_a =$ $(6.19 \pm 1.5) \times 10^{-10}$

$10^{-2}\ E_{SH} =$ 113.6 ± 1.1

$10^{-2}\ E_{S-} =$ 77.4 ± 1.6

Supplementary Table S11 The values of observed absorbance (A_{obs}) at 253 nm as a function of pH for ionization of Boldine **3** in 2% v/v acetonitrile

**pH**	**A_{obs}**	**A_{calc}**	**%RE**
12.48	1.358	1.320	2.811
11.68	1.356	1.316	2.983
11.11	1.291	1.302	-0.858
10.53	1.186	1.255	-5.826
9.80	1.033	1.073	-3.918
9.40	0.944	0.921	2.441
8.83	0.791	0.751	5.047
8.58	0.739	0.709	4.034
8.40	0.698	0.689	1.299
7.96	0.712	0.662	7.041
7.34	0.686	0.649	5.334
6.97	0.670	0.647	3.418
5.85	0.612	0.646	-5.475
5.45	0.618	0.645	-4.438
4.85	0.613	0.645	-5.284
4.21	0.619	0.645	-4.261
3.80	0.622	0.645	-3.758
2.53	0.619	0.645	-4.261

$K_a = (2.74 \pm 0.5) \times 10^{-10}$

$10^{-2} E_{SH} = 64.5 \pm 1.2$

$10^{-2} E_{S-} = 132 \pm 2.1$

Supplementary Data 1 Nonlinear least squares computer program in BASICA

```
30   PRINT "NO. OF PARAMETERS  =  ";
40   INPUT N
50   PRINT "NO. OF POINTS";
60   INPUT K
70   DIM J(K,N),L(N,K),E(K,1),C(K,1),O(K,2),B(K,1),V(1,K),W(N,1)
80  DIM M(N,N),X(N,1),T(N,1),U(1,1),F(N,N),S(N,1)
90   PRINT "NN   =";
100  INPUT NN
105 PRINT "value of cmc =";
106 INPUT C1
190  FOR I=1 TO K
210  READ  Z1
215  O(I,1) =10^(-Z1)
220  NEXT I
230  FOR I= 1 TO K
250  READ  Z2
255  O(I,2) = Z2
260  NEXT I
270  PRINT "INITIAL GUESS VALUE OF A1 =";
280  INPUT A1
290  PRINT "INITIAL GUESS VALUE OF A2 =";
294  INPUT A2
295  IF N=2 THEN 310
296  PRINT "INITIAL GUESS VALUE OF A3 =";
297  INPUT A3
298  IF N=3 THEN 310
299  PRINT "INITIAL GUESS VALUE OF A4 =";
```

```
300  INPUT A4
302  IF N=4 THEN 310
304  PRINT "INITIAL GUESS VALUE OF A5 =";
306  INPUT A5
310  PRINT "INITIAL CONC. OF SUBS. =";
320 INPUT X0
322 PRINT "value of kw =";
323 INPUT KW
330 T(1,1)=A1
340 T(2,1)=A2
342 IF N=2 THEN 360
346 T(3,1)=A3
350 IF N=3 THEN 360
352 T(4,1)=A4
354 IF N=4 THEN 360
356 T(5,1)=A5
360 PRINT "TOTAL # OF ITERATION  =:
370 INPUT K2
380 DATA 6.08,6.33,6.8,7.43,7.73,8.1,8.17,8.26,8.43,8.57,8.83,9,9.15,9.38,9.56,9
.78,9.9,10.11
381 DATA .845,.855,.832,.905,1.05,1.179,1.334,1.294,1.465,1.587,1.721,1.824,1.92
3,2.07,2.131,2.192,2.25,2.21
382 DATA .000145,.000156,.00015
400 PRINT"ERROR CHECK  =";
410 INPUT E0
420 PRINT"TOTAL # OF ITERATION  ="K2
421 PRINT"ERROR CHECK  ="E0
430 GOSUB 722
```

```
440 A1=T(1,1)
450 A2=T(2,1)
452 IF N=2 THEN 470
454 A3=T(3,1)
456 IF N=3 THEN 470
458 A4=T(4,1)
460 IF N=4 THEN 470
464 A5=T(5,1)
470 FOR I=1 TO K
471 V(1,I)=E(I,1)
472 NEXT I
480 U(1,1)=0!
481 FOR I=1 TO K
482 U(1,1)=U(1,1)+V(1,I)*E(I,1)
483 NEXT I
490 FOR I=1 TO N
500 S(I,1)=SQR(U(1,1)*F(I,I)/(K-N))
510 NEXT I
520 PRINT"ITERATION  #  ="K1
530 PRINT"A1    ="T(1,1);"STD.   ="S(1,1)
540 PRINT"A2    ="T(2,1);"STD.   ="S(2,1)
550 IF N=2 THEN 590
560 PRINT"A3    ="T(3,1);"STD.   ="S(3,1)
570 IF N=3 THEN 590
580 PRINT"A4    ="T(4,1);"STD.   ="S(4,1)
585 IF N=4 THEN 590
587 PRINT"A5    =";T(5,1);"STD.  ="S(5,1)
590 PRINT"LEAST SQ. VALUE  ="U(1,1)
```

```
600 PRINT"----------------------------------------------------------------"

610 FOR I=1 TO N

620 IF ABS(W(I,1)/T(I,1))<E0 THEN 640

630 IF ABS(W(I,1)/T(I,1))>E0 THEN 660

640 NEXT I

641 PRINT"DO YOU WANT LSQ";

642 INPUT Y9

650 GOTO 680

660 K1=K1+1

670 IF K1<K2 THEN 430

680 PRINT"TIME        Aobs        Acalcd      % Res. Error"

690 FOR I=1 TO K

700 PRINT O(I,1),O(I,2),C(I,1),100*(O(I,2)-C(I,1))/O(I,2)

710 NEXT I

720 STOP

722 IF NN>2 THEN 792

730 FOR I=1 TO K

740 J(I,1)=(X0/(O(I,1)+A1))*(A3-((A2*O(I,1)+A3*A1)/(O(I,1)+A1)))

750 J(I,2)=X0*O(I,1)/(O(I,1)+A1)

760 IF N=2 THEN 785

765 J(I,3)=X0*A1/(A1+O(I,1))

770 IF N=3 THEN 785

774 J(I,4)=

776 IF N=4 THEN 785

778 J(I,5)=

785 C(I,1)=((A1*A3+A2*O(I,1))/(A1+O(I,1)))*X0

789 E(I,1)=O(I,2)-C(I,1)

790 NEXT I
```

```
791 GOTO 800
792 FOR I=1 TO K
793 J(I,1)=1/(A2+O(I,1))
794 J(I,2)=-A1/((A2+O(I,1))^2)
796 C(I,1)=A1/(A2+O(I,1))
797 E(I,1)=O(I,2)-C(I,1)
798 NEXT I
800  FOR I=1 TO K
801  FOR M=1 TO N
802  L(M,I)=J(I,M)
803  NEXT M
804  NEXT I
806  FOR I=1 TO N
807  FOR J=1 TO N
808  F(I,J)=0!
809  X(I,1)=0!
810  W(I,1)=0!
811  NEXT J
812  NEXT I
813  FOR M=1 TO N
814  FOR JJ=1 TO N
815  FOR I=1 TO K
816  F(M,JJ)=L(M,I)*J(I,JJ) + F(M,JJ)
817  NEXT I
818  NEXT JJ
819  NEXT M
820  FOR KK=1 TO N
825  FOR J= 1 TO N-1
```

```
830  M(KK,J)=F(KK,J+1)/F(KK,1)
840  NEXT J
845  M(KK,N)=1/F(KK,1)
850  IF KK=1 THEN 890
855  I=1
860  R1=F(I,1)
865  FOR J=1 TO N-1
870  M(I,J)=F(I,J+1)-R1*M(KK,J)
875  NEXT J
880  M(I,N)=-R1*M(KK,N)
885  IF KK=2 THEN 915
890  I=2
895  R2=F(I,1)
896  FOR J=1 TO N-1
897  M(I,J)=F(I,J+1)-R2*M(KK,J)
900  NEXT J
905  M(I,N)=-R2*M(KK,N)
910  IF KK=3 THEN 955
915  IF N=2 THEN 1004
920  I=3
925  R3=F(I,1)
930  FOR J=1 TO N-1
935  M(I,J)=F(I,J+1)-R3*M(KK,J)
940  NEXT J
945  M(I,N)=-R3*M(KK,N)
950  IF KK=4 THEN 990
955  IF N=3 THEN 1004
960  I=4
```

```
965  R4=F(I,1)
970  FOR J=1 TO N-1
975  M(I,J)=F(I,J+1)-R4*M(KK,J)
980  NEXT J
983  M(I,N)=-R4*M(KK,N)
986  IF KK=5 THEN 1004
990  IF N=4 THEN 1004
992  I=5
994  R5=F(I,1)
996  FOR J=1 TO N-1
998  M(I,J) =F(I,J+1)-R5*M(KK,J)
1000 NEXT J
1002 M(I,N)=-R5*M(KK,N)
1004 FOR I=1 TO N
1006 FOR J=1 TO N
1008 F(I,J)=M(I,J)
1010 NEXT J
1012 NEXT I
1014 NEXT KK
1020 FOR M=1 TO N
1030 FOR I=1 TO K
1040 X(M,1)=X(M,1)+L(M,I)*E(I,1)
1050 NEXT I
1060 NEXT M
1070 FOR MM=1 TO N
1080 FOR I=1 TO N
1090 W(MM,1)=W(MM,1)+F(MM,I)*X(I,1)
1100 NEXT I
```

```
1110 NEXT MM

1120 FOR I=1 TO N

1130 T(I,1)=T(I,1)+W(I,1)

1140 NEXT I

1150 RETURN

1160 END
```
